# Identification and Characterization of a Leucine-Rich Repeat Kinase 2 (LRRK2) Consensus Phosphorylation Motif

**DOI:** 10.1371/journal.pone.0013672

**Published:** 2010-10-27

**Authors:** Pooja P. Pungaliya, Yuchen Bai, Kerri Lipinski, Vasanti S. Anand, Saurabh Sen, Eugene L. Brown, Brian Bates, Peter H. Reinhart, Andrew B. West, Warren D. Hirst, Steven P. Braithwaite

**Affiliations:** 1 Global Biotherapeutic Technologies, Pfizer Incorporated, Cambridge, Massachusetts, United States of America; 2 Neuroscience Research Unit, Pfizer Incorporated, Princeton, New Jersey, United States of America; 3 Department of Neurology, Center for Neurodegeneration and Experimental Therapeutics, University of Alabama at Birmingham, Birmingham, Alabama, United States of America; Brigham and Women's Hospital, Harvard Medical School, United States of America

## Abstract

Mutations in *LRRK2* (leucine-rich repeat kinase 2) have been identified as major genetic determinants of Parkinson's disease (PD). The most prevalent mutation, G2019S, increases LRRK2's kinase activity, therefore understanding the sites and substrates that LRRK2 phosphorylates is critical to understanding its role in disease aetiology. Since the physiological substrates of this kinase are unknown, we set out to reveal potential targets of LRRK2 G2019S by identifying its favored phosphorylation motif. A non-biased screen of an oriented peptide library elucidated F/Y-x-T-x-R/K as the core dependent substrate sequence. Bioinformatic analysis of the consensus phosphorylation motif identified several novel candidate substrates that potentially function in neuronal pathophysiology. Peptides corresponding to the most PD relevant proteins were efficiently phosphorylated by LRRK2 *in vitro*. Interestingly, the phosphomotif was also identified within LRRK2 itself. Autophosphorylation was detected by mass spectrometry and biochemical means at the only F-x-T-x-R site (Thr 1410) within LRRK2. The relevance of this site was assessed by measuring effects of mutations on autophosphorylation, kinase activity, GTP binding, GTP hydrolysis, and LRRK2 multimerization. These studies indicate that modification of Thr1410 subtly regulates GTP hydrolysis by LRRK2, but with minimal effects on other parameters measured. Together the identification of LRRK2's phosphorylation consensus motif, and the functional consequences of its phosphorylation, provide insights into downstream LRRK2-signaling pathways.

## Introduction

Parkinson's disease (PD) is a neurodegenerative disorder that is associated with progressive loss of dopaminergic neurons in the substantia nigra. Demise of dopaminergic neurons leads to the clinical features of PD which include bradykinesia, rigidity, and resting tremor [1]. Though most PD cases are sporadic, there are several genetic determinants such as mutations in *SNCA*, *Parkin*, *PINK1*, *DJ-1* and *LRRK2* (reviewed in [2]). Linkage analysis studies have shown that missense mutations in leucine-rich repeat kinase 2 (*LRRK2*) lead to an autosomal late-onset form of PD [3], [4]. LRRK2 is a 286 kDa cytoplasmic protein with GTPase and kinase domains as well as multiple protein-protein interaction domains. Mutations have been identified throughout the protein with the most common mutation, G2019S, residing in its kinase domain [5], [6]. *In vitro* data has suggested that the G2019S substitution augments kinase activity by at least 2-fold [7]–[9], therefore making the study of LRRK2's kinase activity important for understanding disease aetiology and for development of therapeutics.

PD is characterized pathologically by presence of Lewy bodies composed of α-synuclein and degeneration primarily of dopaminergic neurons. Several cellular mechanisms underlie these pathological features including mitochondrial dysfunction, protein aggregation, oxidative stress, and synaptic dysregulation [10]–[14]. LRRK2 has been implicated in many of these pathophysiological functions. Patients carrying LRRK2 mutations develop pathological inclusions composed of α-synuclein and ubiquitin aggregates within dopaminergic neurons of the substantia nigra [4], and LRRK2 can potentiate α-synuclein pathology in transgenic mouse models [15]. *Drosophila* expressing mutant forms of LRRK2 have higher levels of hydroxyl-free radicals than dLRRK2 null and heterozygote flies suggesting a function for LRRK2 in oxidative stress [16]. A fraction of LRRK2 co-localizes in synaptic vesicles and overexpression of LRRK2 mutants impairs synaptic vesicle endocytosis [17]. These studies suggest a role for LRRK2 in the pathogenic pathways of PD, but the exact pathways linking the kinase to these functions remain unknown. Fundamental to addressing these questions is the identification of LRRK2's physiological substrate(s).

In this study, two oriented degenerate peptide library scans were employed to identify a LRRK2 G2019S phosphorylation motif as F/Y-x-T-x-R/K. This motif resides within a number of proteins including LRRK2 itself at Thr1410. This Thr residue is found in the ROC domain of LRRK2 and was demonstrated to be a site of autophosphorylation by mass spectrometry. Potential functional relevance of this phosphorylation was evidenced by the finding that a T1410A mutation reduced GTPase activity but not GTP binding, autophosphorylation, or kinase activity. The identification of this motif and potential substrates suggests mechanisms by which LRRK2 can function in PD relevant pathways.

## Methods

### Materials

Biotinylated peptide substrates were purchased from Anaspec, Inc. Positional scanning peptide library was designed and prepared as described by Turk et al. [18]. Peptide concentration was calculated based on the molar absorptivity of the peptides at 280 nm. Recombinant GST-tagged LRRK2 protein 970-2527 (wildtype, G2019S, R1441C, T1410A (G2019S), and D1994A) were obtained from Invitrogen. ATP and GTP were obtained from Sigma. Full length WT-LRRK2 pCMV6-XL5 was purchased from Origene (NM_198578.2). PCR was used to add a 5′ KOZAK sequence, 5′ HA tag, and 3′ Myc tag. All point mutations including G2019S, D1994N, K1347A, and T1410A were inserted using Quickchange site-directed mutagenesis. Antibodies utilized were rabbit phospho-threonine-x-arginine (Cell Signaling Technology), GST-HRP (GE Healthcare), rat HA-HRP (Roche Applied Science), mouse Flag M2-HRP (Sigma), and mouse actin-HRP (Sigma). A rabbit polyclonal phospho-specific antibody against LRRK2 Thr 1410 was produced by 21^st^ Century Biochemicals using a KLH-coupled synthetic phosphopeptide corresponding to residues surrounding Thr 1410 of human LRRK2 (acetyl-THPHFM[pT]QRALYLAC-amide).

### Cell culture, transfection, and immunoprecipitation

HEK-293FT cells (Invitrogen) were cultured in complete Dulbecco's Modified Eagle Medium and maintained at 37°C in 5% CO_2_. Transient transfections were performed using FuGENE HD (Roche Applied Science). For biochemical experiments HEK-293FT cells were cultivated for 72 hours. Lysates were prepared on ice with 20 mM Tris, pH 8.0, 135 mM NaCl, 1 mM MgCl_2_, 0.1 mM CaCl_2_, 10% glycerol, 1% NP-40, 50 mM β-glycerol phosphate, 1 µg/mL leupeptin, 100 µg/mL phenylmethylsulfonyl fluoride, 1 mM sodium fluoride, 1 µg/mL aprotinin, and phosphatase inhibitor cocktails 1 and 2 (Sigma). Protein concentrations in the resulting supernatants were quantitated using standard BCA protein assay (Pierce). For immunoprecipitations, 2.5 mg lysates were incubated with EZview Red Anti-HA affinity gel (Sigma) overnight at 4°C. Precipitated immunocomplexes were washed in the lysis buffer and either assayed for activity or subjected to immunoblot analysis by boiling samples in LDS sample buffer with reducing agent (Invitrogen). Samples were separated on 3–8% NuPAGE Tris-Acetate gels (Invitrogen), transferred onto nitrocellulose membranes (Invitrogen), and processed for western analysis with anti-pT1410 LRRK2 or anti-HA antibodies.

### Kinase assay with biotinylated peptide substrates

Kinase assays were performed with 5 nM recombinant LRRK2 G2019S or LRRK2 D1994A (Invitrogen), 200 µM biotinylated peptide, 1 µCi of [γ-^33^P] ATP, and 200 µM ATP in a total volume of 25 µL of kinase buffer (25 mM MOPS, pH 7.2, 12.5 mM β-glycerophosphate, 25 mM MgCl_2_, 5 mM EGTA, 2 mM EDTA, 0.002% BSA, and 0.25 mM DTT). Kinase reactions were setup such that LRRK2 protein and [^33^P] ATP were pre-incubated at 30°C for 30 minutes followed by addition of the peptide substrate for 30 additional minutes. In indicated experiments, HA-tagged LRRK2 protein immunoprecipitated from HEK-293FT cells was used. Protein concentration and incubation time were optimized to obtain approximately 10% peptide phosphorylation. Reactions were terminated by adding 12.5 µL 7.5 M guanidine hydrochloride and 5 µL of the reaction was spotted onto SAM^2^ Biotin Capture Membrane (Promega) [19]. All reactions were done in triplicate. Washes were performed as described by Turk et al. and the membrane squares were air dried for 1 hour [18]. Finally, 5 mL of Opti-Fluor (Perkin-Elmer) was added to each membrane square and emissions were measured in a scintillation counter (Beckman-Coulter).

### Autophosphorylation assay

5 nM LRRK2 protein (wildtype, G2019S, or T1410A (G2019S)) was incubated for 30 min at 30°C with 1 µCi of [γ-^33^P] ATP and 200 µM ATP in kinase buffer as described above. As controls, reactions were performed in the absence of LRRK2 protein or ATP. Where indicated, HA-tagged LRRK2 protein immunoprecipitated from HEK-293FT cells was used. Reactions were stopped by adding LDS sample buffer with reducing agent (Invitrogen) followed by boiling. Samples were separated on 7% NuPAGE Tris-Acetate gels (Invitrogen) and transferred onto nitrocellulose membranes (Invitrogen). The nitrocellulose membranes were probed with anti-pT1410 LRRK2, anti-phospho-threonine-x-arginine, anti-HA, or anti-GST antibodies. In some cases, an autoradiogram was generated by exposure to film at −80°C overnight and band intensities were quantified by densitometry.

### GTP hydrolysis assay

The GTPase activity of LRRK2 was measured using the Colorimetric GTPase assay kit (high sensitivity) according to the manufacturer's instructions (Innova Biosciences) and by PEI-cellulose Thin layer chromatography (TLC) plate analysis as previously described [20]. For the colorimetric GTPase assay, components of the reaction included 5 nM LRRK2 protein (wildtype, G2019S, R1441C, or T1410A) in a buffer containing 50 mM Tris, pH 7.5, 2.5 mM MgCl_2_, 0.5 mM GTP, 10 µM ATP, 0.1 mM DTT, and 0.00005% BSA. As controls, some reactions contained no protein. Total volume for each reaction was 200 µL and each reaction was done in triplicate. Each reaction was incubated at 30°C for 0, 15, 30, or 60 min and was stopped by the addition of 50 µL Gold mix (P_i_ ColorLock Gold and Accelerator). After 2 min room temperature incubation, 20 µL stabilizer was added followed by incubation at room temperature for 30 min. After 30 min, absorbance was recorded at 635 nm using a SpectraMax 250 plate reader (Molecular Devices). For GTP hydrolysis assay using TLC plates, HA-tagged LRRK2 proteins (wildtype, G2019S, K1347A, T1410A (G2019S), T1410D (G2019S), D1994N) were incubated at 30°C for 60 min in a buffer with 50 mM Tris-HCl, pH 8.0, 2.5 mM MgCl_2_, 0.5 mM GTP, 10 µCi [α-^32^P] GTP, 10 µM ATP, 0.1 mM DTT, and 0.00005% BSA. Reactions were stopped with 0.5 M EDTA and 1 µL was separated by PEI-cellulose TLC plate using 0.5 M KH_2_PO_4_, pH 3.4 buffer. Plates were dried for 30 min and radioactive bands detected by autoradiography. Background activity was measured using extracts from vector only transfected control cells.

### GTP-binding assay

GTP-binding assay was performed as previously described [20], [21]. Briefly, GTP-agarose beads (Sigma) were pre-treated with 1X TBS containing 0.01% BSA for 1 h at 4°C to block non-specific binding sites. Then, the beads were equilibrated in a binding buffer containing 25 mM MOPS, pH 7.2, 12.5 mM β-glycerolphosphate, 25 mM MgCl_2_, 5 mM EGTA, 2 mM EDTA, 0.002% BSA, and 0.25 mM DTT. Cell lysates containing transfected HA-tagged LRRK2 (wildtype, G2019S, K1347A, or T1410A (G2019S)) were incubated with the GTP-agarose for 1 h at 4°C. Controls were performed with no LRRK2 protein. After incubation, competition experiments were performed by adding 10 mM GTP for an additional 2 h at 4°C. Beads were washed with binding buffer three times followed by boiling in LDS sample buffer with reducing agent (Invitrogen). Samples were separated on 7% NuPAGE Tris-Acetate gels (Invitrogen) and transferred onto nitrocellulose membranes (Invitrogen). The nitrocellulose membranes were probed with anti-HA antibody.

### LanthaScreen™ Kinase Assay

LanthaScreen™ assays were performed as previously described [7]. Briefly, LRRK2 protein (wildtype, G2019S, or T1410A (G2019S)) was diluted 1∶2 for 15 points starting at 0.15 mg/ml. Assay reagents were made up to a final of 100 nM fluorescein-LRRKtide and 50 µM ATP in 1X assay buffer 50 mM HEPES pH 7.5, 3 mM MgCl2, 0.01% Brij, and 2 mM DTT. The reactions proceeded for 1 hr at room temperature in the dark. Reactions were stopped by adding 10 µl of 3 mM EDTA and 1 nM terbium-labeled anti-p-LRRKtide antibody (final concentrations) in 1X TR-FRET dilution buffer (20 mM Tris, pH 7.5, 0.02% sodium azide, and 0.01% NP-40 alternative). Plates were incubated for 1 hr at room temperature in the dark. Finally, plates were read on Envision plate reader (Perkin Elmer) and emission ratios at 525 nm and 495 nm were determined.

### In-gel digestion and peptide identifications by LC-MS/MS

Recombinant LRRK2 G2019S protein was subjected to an *in vitro* autophosphorylation assay as described above for 1 hour. The reaction was separated on a 3–8% NuPAGE Tris-Acetate gel (Invitrogen) followed by Coomassie blue staining. The LRRK2 G2019S band was excised and subjected to robotic in-gel digestion by trypsin as previously described [22]. The peptide samples were loaded onto a reversed-phase C_18_ capillary column (5 µm, 200 Å, 75 µm×10 cm) and coupled to an LTQ-Orbitrap XL mass spectrometer (ThermoFisher Scientific, San Jose, CA). Peptides were separated at a flow rate of 0.2 µL/min using a 90 min linear gradient ranging from 2% to 40% B (mobile phase A: 0.1% formic acid/2% CH_3_CN; mobile phase B: 90% CH_3_CN/0.1% formic acid). Electrospray voltage was 1.8 kV. For the analysis of unlabeled peptides, the instrument method consisted of a full MS scan (scan range 375–1550 m/z, with 30K FWHM resolution @ m/z = 400, target value 2×10^6^, maximum ion injection time of 500 ms) followed by data-dependent CID scans of the six most intense precursor ions. Peptide precursor ions were selected with an isolation window of 2.5 Da, and a target value of 1×10^5^. Dynamic exclusion was implemented with a repeat count of 2 and exclusion duration of 75 sec. The normalized collision energy was set to 35%.

The MS/MS spectra were searched against human LRRK2 sequence using Bioworks 3.3.1 SP1 with SEQUEST search algorithm (ThermoFisher Scientific, Version 28). For SEQUEST searches, the mass windows for precursor and fragment ions were 30 ppm and 1 Da respectively. The search database was the International Protein Index (IPI), Human division, version 3.56, concatenated with a reversed version of the same database, to estimate the false discovery rate. Search space was limited to fully tryptic peptides with up to 2 missed cleavages. The fixed modification carbamidomethyl (C) +57.02146 Da was applied. Four differential modifications were allowed: Phosphophorylation (STY) +79.9663 Da, Oxidation (MW) +15.9949 Da, and double Oxidation (W) +31.9898. A total of 2 differential modifications of any one type, and 3 total modifications were allowed for any single peptide. The top-ranked peptide-spectrum match (PSM) from each query spectrum was retained for further analysis. Identified peptides were mapped to their cognate proteins in the source database. Phospho-peptides that mapped to LRRK2 were ordered by the SEQUEST XCorr score, and in cases where the same phospho-peptide was identified multiple times, the top-scoring PSM was retained.

### Sequence logo analysis

The position-specific scoring matrix (PSSM) logo was generated by R scripts. Some R code was adapted from the Bioconductor seqLogo package for DNA alignments [23]. Sequence logos are a graphical representation of sequence alignments first developed by Schneider and Stephens [24].

### Native PAGE protein analysis

Protein expression analysis by native PAGE was performed as previously described [25]. Transfected cells were gently harvested by a cell scraper, divided in half, and pelleted by centrifugation at 200×*g* for 5 min at 4°C. Cell pellets were resuspended with either native lysis buffer (phosphate-buffered saline (PBS), pH 7.4, 1x protease and phosphatase inhibitors (Roche Applied Science), lysed by four cycles of freezing and thawing, and analyzed on a 3–12% bis-tris Blue native polyacrylamide gel (Invitrogen) or lysates combined with SDS-lysis buffer (PBS, pH 7.4, 1% SDS, 1xprotease and phosphatase inhibitors (Roche Applied Science) and sonicated with 10% power for 10 s on a Branson dismembranator and analyzed on a 7% Tris acetate SDS gel (Invitrogen). Prior to PAGE, lysates were centrifuged for 10 min at 20,000×*g*, and protein content in supernatant was determined by a BCA assay (Pierce). Proteins resolved in acrylamide were transferred to PVDF membranes (Immobilon P, Millipore) at 4°C overnight to ensure complete transfer of high molecular weight species. In most experiments, PVDF membranes were blocked with 5% nonfat milk in TBS-T (Tris-buffered saline Tween-20) for 1 h and incubated overnight in blocking buffer supplemented with anti- HA antibody (Sigma) and developed using electrochemical luminescence substrate (Pierce).

## Results

### Identification of a consensus LRRK2 G2019S phosphorylation site

**Figure 1:**
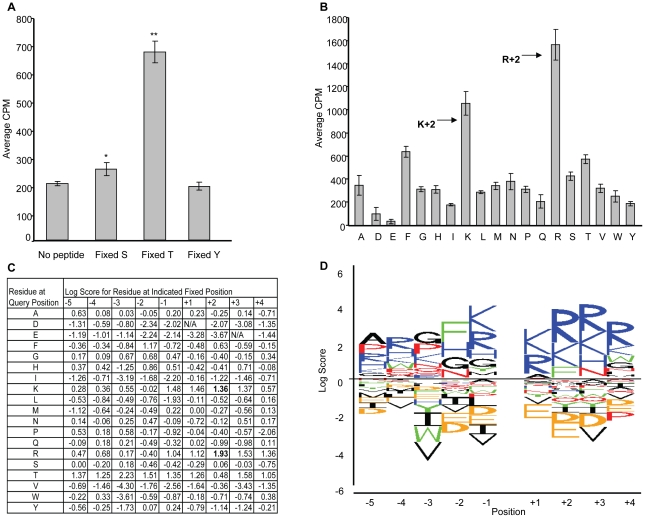
Primary peptide positional screening scan for LRRK2 G2019S. (A) To determine which phosphoacceptor site LRRK2 G2019S prefers, fixed phosphoacceptor pools or no peptide were incorporated into a kinase assay with recombinant LRRK2 G2019S and ^33^P[ATP]. Fixed S = Y-A-X-X-X-X-X-S-X-X-X-X-A-G-K-K(LC-Biotin)-NH2, Fixed T = Y-A-X-X-X-X-X-T-X-X-X-X-A-G-K-K(LC-Biotin)-NH2, and Fixed Y = Y-A-X-X-X-X-X-Y-X-X-X-X-A-G-K-K(LC-Biotin)-NH2 where X is a degenerate position (adapted from [18]). Threonine was the favored phosphoacceptor when compared to serine and tyrosine (**p<0.001). Serine was phosphorylated greater than no peptide control (*p<0.05). (B) Results of the peptide positional scan demonstrates that Arg/Lys +2 is favored. Shown are peptide pools where Z is a fixed residue at +2 ie X-X-X-X-X-S/T-X-Z-X-X. (C) Table listing the log scores for each peptide pool. Arg and Lys at the +2 position have highest log scores (bold). Log score calculated as log2[(counts)/(mean counts for all peptides at the same position)] [31]. N/A denotes peptide pools where CPM counts were equal to or less than no peptide control. (D) Sequence logo representation for peptide positional scan highlights favored (above line) and disfavored residues (below line). Residues in each stack are sorted by log score with the height of each letter proportional to the absolute value of the residue's log score (adapted from [31]). Position 0 is the fixed Ser/Thr phosphoacceptor site. Residue color code: nonpolar = black, aromatic = green, polar uncharged = red, positively charged = blue, negatively charged = orange.

Kinase specificity is often achieved by recognition of amino acids surrounding the site of phosphorylation, therefore we attempted to identify sequences that are most critical for LRRK2's optimal activity. Biotinylated oriented degenerate peptide libraries synthesized as 10 mers with a fixed phosphoacceptor were used [18], [26]–[28]. First, to determine whether LRRK2 G2019S preferred a particular phosphoacceptor residue, peptide substrates were utilized with either Ser, Thr, or Tyr fixed at the central residue with flanking degenerate sequence (x-x-x-x-x-S/T/Y-x-x-x-x) [18]. Threonine was the favored phosphoacceptor when compared to Ser and Tyr (*p*<0.001) (Figure 1A), although peptides with Ser as the phosphoacceptor were also phosphorylated significantly above negative controls (*p*<0.05).

**Figure 2:**
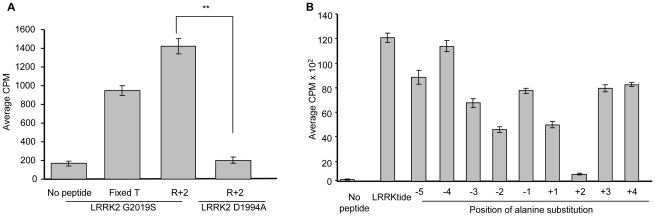
LRRK2 G2019S prefers Arg at the +2 position in its substrates. (A) LRRK2 G2019S or a kinase dead mutant (LRRK2 D1994A) was incubated with either no peptide (negative control), fixed Thr phosphoacceptor pool (positive control), or Arg +2 peptide pool. ^33^P incorporation by kinase dead mutant is equivalent to no peptide control showing phosphorylation of Arg +2 peptide pool is LRRK2 G2019S kinase dependent. Reduced activity was detected between LRRK2 D1994A and LRRK2 G2019S (**p<0.001). (B) Alanine scanning of known LRRK2 substrate LRRKtide (TKPRLGRDKYKTLRQIRQ-biotin) also shows preference for Arg at +2. Alanine was substituted for each residue from −5 to +4. Note reduction of phosphorylation upon substitution of Arg +2 compared to wildtype LRRKtide.

Next, specificity to surrounding residues was determined using positional scanning of the biotinylated oriented peptide library with peptide substrates in which Thr was fixed as the phosphoacceptor. Screening of peptides with fixed residues at the +2 position with LRRK2 G2019S revealed preference for basic amino acids Arg and Lys (Figure 1B). These results are in line with studies using recombinant HEK-293 purified LRRK2 G2019S protein [29]. To ensure that selection of Arg at the +2 position was not due to a contaminating kinase, we also investigated kinase dead LRRK2 D1994A [30]. Phosphorylation of peptides containing Arg +2 was shown to be LRRK2 G2019S kinase domain dependent with no phosphorylation by LRRK2 D1994A (Figure 2A).

Quantitation of preference across all residues was calculated using the “ratio to mean” and “log scores” [31] for each peptide pool at each fixed position (Figure 1C). Arg +2 had a high log score (1.93) indicating that it is a strongly favored residue. Several fixed Thr peptide pools, at −3, −2 and +3 positions, also appeared to be favored. As Thr is a phosphoacceptor we tested peptides that had phosphothreonine at each of the nine fixed positions and no preference was detected (data not shown). These data indicated that the increased signal at these Thr residues in the original screen is through their additional phosphorylation rather than a relevant selection. Furthermore, the log scores were input as a matrix in a sequence logo analysis [24], [32] to depict the relative preference for each amino acid (Figure 1D). This first analysis revealed the optimal LRRK2 G2019S consensus sequence to be A-R-G-F-K-T-K-R-R-R.

**Figure 3:**
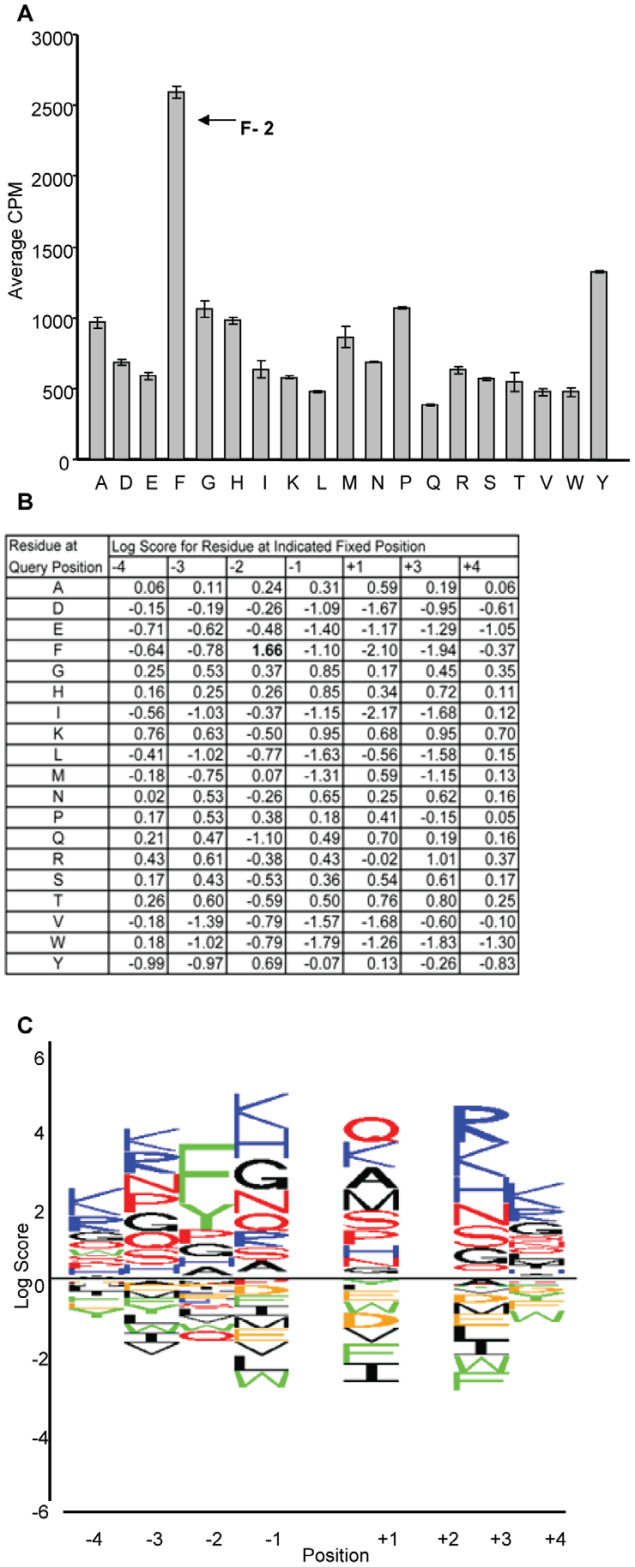
Secondary peptide positional scan supports selection for Phe at the −2 position. (A) Each peptide pool has threonine fixed as the phosphoacceptor and Arg fixed at the +2 position. Each bar represents one peptide pool. Shown are peptide pools where Z is a fixed residue at −2 ie X-X-X-Z-X-T-X-R-X-X where X is a degenerate position (adapted from [18]). (B) Table lists the log scores for each peptide pool. Phe at the −2 position has the highest log score (bold). (C) Sequence logo representation for secondary peptide scan. Position 0 is Thr and position +2 is Arg.

We sought to further refine the motif by fixing Thr as the phosphoacceptor and Arg at +2 position (x-x-x-x-T-x-R-x-x) in a new peptide library. Phenylalanine (Phe) at the −2 position demonstrated the strongest preference (Figure 3A–3C), in line with results from the first scan (Figure 1). These data extended the motif to favor Phe at −2 and Arg at +2. The second analysis therefore refined the optimal LRRK2 G2019S recognition sequence to K-K-F-K-T-Q-R-R-K. Consideration of secondary preferences at the +2 and −2 positions was also considered. Tyrosine is an aromatic residue, like Phe, and showed the second strongest signal at the −2 position, therefore may also be considered to influence selection. Similarly Lysine at the +2 position was highly selected in the original scan (Figure 1C, 1D) indicating this may also be a relevant contributor to LRRK2 recognition. Therefore a less stringent consensus motif of F/Y-x-T-x-R/K was also identified from these studies.

LRRK2 has been shown to efficiently phosphorylate moesin at Thr 558, and a peptide, LRRKtide, derived from it [33]. Therefore to confirm the major selections from the positional scanning technique alanine scan substitutions were performed. The alanine scan demonstrated that substitution of Arg +2 resulted in the most significant decrease in phosphorylation efficiency followed by substitution at the Tyr −2 position (Figure 2B). Therefore the critical nature of the peptides at +2 and −2 positions were confirmed.

**Figure 4:**
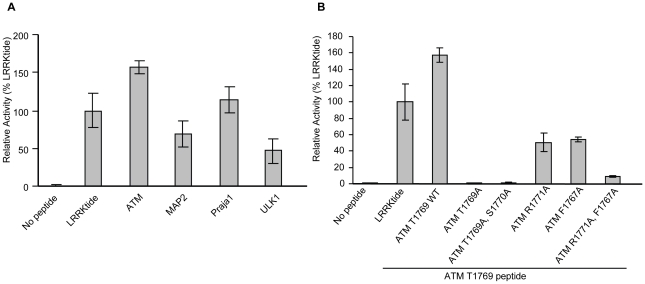
LRRK2 G2019S phosphorylates predicted peptide substrates. (A) Sequences for ATM, MAP2, Praja1, and ULK1 were synthesized as listed in Table 1. All peptides were biotinylated and incorporated into kinase assays with 5 nM LRRK2 G2019S. (B) Various mutations of biotinylated ATM peptide: AYLQPFRTSRKKFLE were incorporated into kinase assays with LRRK2 G2019S to demonstrate that Phe −2 and Arg +2 confer selection of the ATM peptide.

Database searches using a motif scanning algorithm [34] (scansite.mit.edu) identified candidate substrates for LRRK2 G2019S that contain sites that most resemble the sequences derived from the two peptide scans (Table 1). The ability of LRRK2 G2019S to phosphorylate peptides derived from four of these, ATM, MAP2, Praja1, and ULK1, was assessed due to their potential physiological relevance to the PD phenotype. All of the peptides were phosphorylated by LRRK2 G2019S with ATM and Praja1 phosphorylated at levels similar to LRRKtide (Figure 4A). Moreover, mutations at Phe −2 and Arg +2 of the ATM peptide showed a reduction in phosphorylation (Figure 4B) suggesting that these sites are required for selection.

**Table 1:** Potential LRRK2 substrates.

Protein	Site	Sequence	Score
Serine-protein kinase ATM (Ataxia telangiectasia mutated)	1769	AYLQPFRTSRKKFLE	0.13
Microtubule-associated protein 2 (MAP 2)	1433	RKEKPFKTGRGRIST	0.14
ADAM 29 precursor (A disintegrin and metalloproteinase domain 29)	161	SEEKQFSTMRSGFMQ	0.15
Fanconi anemia group C protein (FACC protein)	171	NHLNGFNTQRRMAPE	0.16
Pancreatic lipase	83	ISGSNFKTNRKTRFI	0.17
Cartilage leucine-rich protein	350	FRSCKFPTKRSKKAG	0.18
Homeobox protein Nkx-3.2	261	FQNRRYKTKRRQMAA	0.18
Homeobox protein Nkx-3.1	179	FQNRRYKTKRKQLSS	0.19
Chromodomain-helicase-DNA-binding protein 2	197	RVKKQPKTQRGKRKK	0.19
Serine/threonine-protein kinase WNK4	1196	LSKGSFPTSRRNSLQ	0.20
Protein C21orf5	2256	TLNGAFKTQRQLPAD	0.22
Lactase-phlorizin hydrolase	1914	KRSKQGKTQRSQQEL	0.23
Splicing factor arginine/serine-rich 11 (p54)	332	TPPKSYSTARRSRSA	0.23
Ubiquitin protein ligase Praja1	74	RSRSPFSTTRRSWDD	0.23
5-oxoprolinase	622	DMLRAFGTSRQARGL	0.24
Syntabulin	560	TVLWAFSTQRGGTDP	0.24
Serine/threonine-protein kinase ULK1	456	QSPTQFQTPRSSAIR	0.24
Cylicin II	45	PKPQRPGTKRRSKPS	0.24
Microtubule plus end-directed kinesin motor 3A	684	KGKARPKTGRRKRSA	0.24
Ras-related protein Rab-21	200	NGSSQPGTARRGVQI	0.25
Transforming growth factor beta 1 precursor (TGF-beta 1)	241	VDINGFTTGRRGDLA	0.25
Dual specificity phosphatase Cdc25A	288	EESPPGSTKRRKSMS	0.25
INT-2 proto-oncogene protein precursor (Fibroblast growth factor-3)	169	RPRRGFKTRRTQKSS	0.25
Inhibitor of growth protein 1	326	PKEKKAKTSKKKKRS	0.25
Serine/threonine-protein kinase Nek3	288	IKNSKHNTPRKKTNP	0.25

The top-scoring predicted hits that match the LRRK2 phosphorylation consensus were identified using Scansite (scansite.mit.edu).

### LRRK2 autophosphorylates at consensus phosphorylation sites

**Figure 5:**
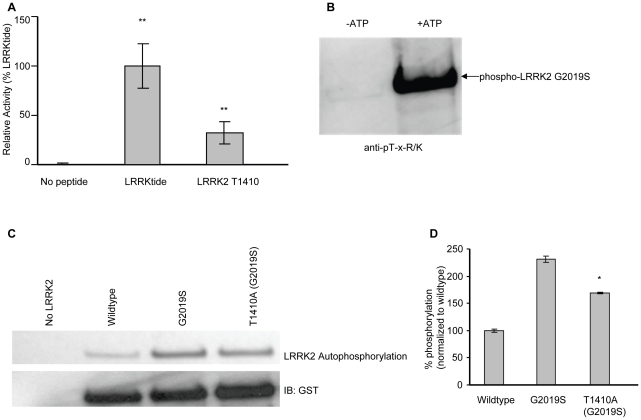
LRRK2 autophosphorylates at T-x-R/K *in vitro*. (A) Kinase assays were performed with LRRKtide and LRRK2 T1410 peptide with LRRK2 G2019S (**p<0.001 in comparison with no peptide). The LRRK2 T1410 peptide sequence was STHPHFMTQRALYLA (B) Autophosphorylation assay using recombinant LRRK2 G2019S with and without ATP confirms that autophosphorylation occurs at T-x-R/K sites. Reaction products were resolved by SDS-PAGE and immunoblotted with anti-pT-x-R/K antibody. (C) Recombinant LRRK2 protein wildtype, G2019S, and T1410A (G2019S) were assayed for autophosphorylation activity. Activities detected by ^33^P autoradiogram (top panel). Equal protein loading shown by anti-GST western blot (bottom panel). (D) Experiments performed in triplicate, quantified, and normalized (*p<0.05 compared to G2019S).

Since LRRK2 autophosphorylation has been identified in multiple studies [35]–[37], we investigated whether the identified consensus motif is also present in LRRK2 itself. The only match was at Thr1410 and a peptide generated to include this site was phosphorylated by LRRK2, indicating its relevance (Figure 5A). Confirmation and functional characterization of T1410 phosphorylation was therefore further investigated in LRRK2 proteins.

To investigate if LRRK2 G2019S autophosphorylates at T-x-R/K sites, we performed *in vitro* kinase assays with LRRK2 G2019S protein and immunoblotted with an antibody that recognizes pT-x-R/K, indicating phosphorylation of this motif (Figure 5B). LRRK2 G2019S that had undergone autophosphorylation *in vitro* was further analyzed by liquid chromatography and tandem mass spectrometry (LC-MS/MS). Multiple phosphorylated residues including T1410 were detected that conformed to the [T/S]-x-[R/K] motif (Supplemental Table S1). Among the [T/S]-x-[R/K] motif sites phosphorylated were residues in the ROC domain (S1345), the COR domain (S1536, T1612), and in the kinase domain (S1913). When LRRK2 T1410A protein in the G2019S background was used in an autophosphorylation assay, the amount of incorporated ^33^P was reduced compared to G2019S (*p<0.05), indicating the loss of the T1410 phosphoacceptor site (Figure 5C, 5D).

**Figure 6:**
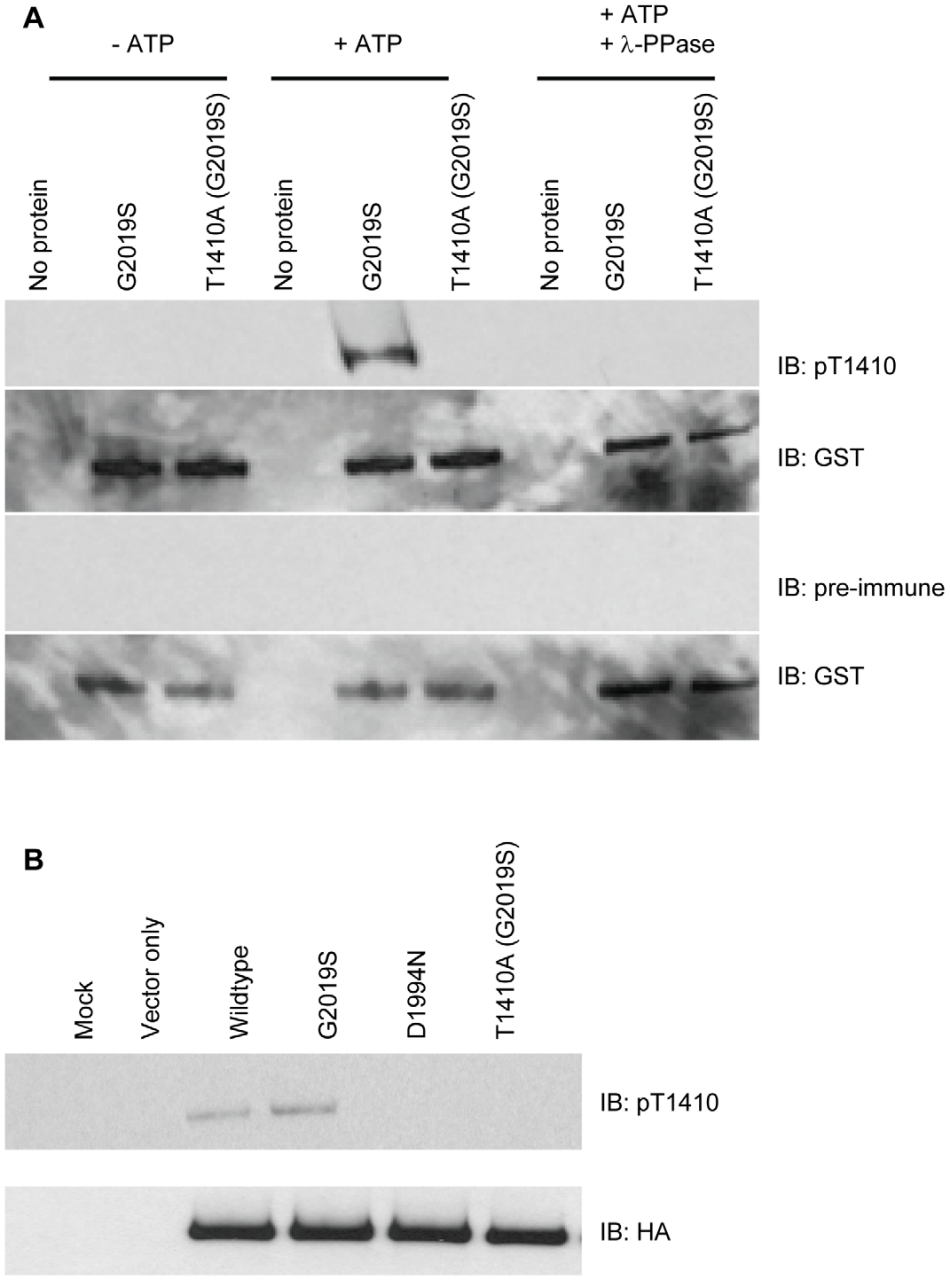
Characterization of LRRK2 autophosphorylation at T1410. (A) Using LRRK2 G2019S and LRRK2 T1410A (G2019S) recombinant proteins in autophosphorylation assays, LRRK2 pT1410 antibody was shown to be site specific (lane 5 versus lane 6) and phospho-specific (lanes 2 and 8 versus lane 5). Phosphorylated LRRK2 G2019S is detected with the LRRK2 pT1410 antibody but not with pre-immune serum. (B) Full length LRRK2 constructs (as indicated) were transfected into HEK-293FT cells, lysed on the plate, and immunoprecipitated with anti-HA affinity gel. Reaction products were analyzed by SDS-PAGE followed by western analysis with pT1410 and HA antibodies. Autophosphorylation was detected at T1410 in wildtype and G2019S but not in kinase dead LRRK2 D1994N or LRRK2 T1410A (G2019S). Equivalent LRRK2 protein levels were confirmed by anti-HA immunoreactivity.

To determine if LRRK2 G2019S autophosphorylates at T1410 in cells, a phosphospecific antibody was generated and utilized. Recombinant LRRK2 T1410A protein was used to confirm that the antibody was site specific and phosphospecific (Figure 6A). Transient transfections of HEK293 cells demonstrated that LRRK2 and LRRK2 G2019S autophosphorylate, with a kinase dead version (D1994N) not autophosphorylating at this site (Figure 6B). Together these data demonstrate that LRRK2 autophosphorylates at T1410 *in vitro* and in cells confirming and extending on data obtained previously using mass spectrometry [35]–[37].

### Mutation of T1410 affects GTPase activity but not GTP binding, autophosphorylation, or kinase activity

**Figure 7:**
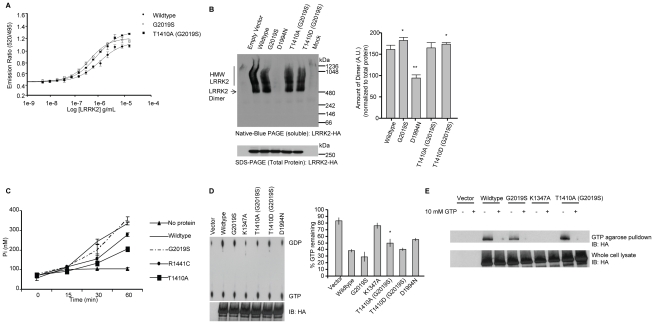
Functional characterization of modulation of LRRK2 at T1410. (A) A LanthaScreen assay was used to assess LRRK2 T1410A kinase activity. Titration of wildtype, G2019S, and T1410A (G2019S) LRRK2 proteins demonstrate that mutation T1410A does not affect phosphorylation of LRRKtide. (B) To investigate dimer formation HEK-293T cells were transfected with the indicated LRRK2 constructs. Cell pellets were split equally for lysis by freeze/thaw cycles directly in PBS or lysis with 1% SDS and PBS with sonication, and 10 ug of protein lysate was loaded onto a native gel (3–12% Bis-Tris) or an SDS gel (7% Tris acetate-SDS), respectively. LRRK2 complexes were visualized with the anti-HA antibody by Western blot with standard ECL. Dimer-sized structures are evident (signal from 480 to 550 kDa), monomeric LRRK2 (278 kDa) is only visible by overexposure (Supplemental Figure S1, [25]).Western blots representative of five independent experiments are shown. Normalization of the LRRK2 dimer to total LRRK2 protein (SDS-solubilized) was done using densitometry analysis. **p<0.001, *p<0.05 n.s., nonsignificant in comparison with wild-type LRRK2, assessed by unpaired Student-Newman-Keuls Multiple Comparison Test. (C) Investigation of LRRK2 GTPase activity shows that LRRK2 T1410A has reduced GTPase activity compared to wildtype. A colorimetric assay was used to assess GTPase activity for LRRK2 wildtype, G2019S, R1441C, and T1410A. The assay was performed in triplicate. Reduced activity was seen between T1410A and wildtype at time points 15, 30, and 60 minutes (*p*<0.01). (D) Full length HA-tagged LRRK2 proteins were immunoprecipitated and assayed for GTP hydrolysis using thin-layer chromatography. Reduced activity was seen between LRRK2 T1410A and wildtype (*p<0.05). (E) GTP binding was not affected by T1410 mutation. Full length LRRK2 proteins were precipitated from cell lysates using GTP-agarose beads. In some cases, 10 mM GTP was added for competition. LRRK2 K1347A was included as a negative control since this mutant does not bind GTP. Reaction products were resolved by SDS-PAGE and immunoblotted with anti-HA antibody (upper panel). LRRK2 expression was equivalent across all transfections as indicated by immunoblotting whole cell lysates with anti-HA antibodies (lower panel).

Next we studied the consequences of mutating LRRK2 at T1410 on its functional properties. To assay whether T1410 influences kinase activity we performed assays with LRRKtide as the substrate (Figure 7A). As expected, LRRK2 G2019S was more active than wild-type LRRK2, but no significant difference in kinase activity was detected between LRRK2 G2019S and T1410A (G2019S) with LRRKtide. These data are consistent with the notion that LRRK2 T1410A does not affect kinase activity on an exogenous substrate [35].

Dimerization has been postulated to be necessary for LRRK2 kinase activity and the GTPase domain important in mediating dimerization [25], [38]–[40]. Therefore we investigated the effect of mutation of T1410 on dimerization of LRRK2 in HEK293 cells. Native-PAGE analysis of full length phosphomimetic LRRK2 T1410D compared to wildtype demonstrated slightly increased dimer formation, whereas T1410A mutation had no effect (Figure 7B). These data suggest that modification at T1410 may have a subtle effect on dimerization.

Because T1410 is located within the GTPase domain it led us to investigate whether it affected the GTP hydrolysis activity of LRRK2. Therefore, a malachite green based colorimetric assay was utilized to measure inorganic phosphate released by LRRK2 [41]–[44]. To assess proper GTP hydrolysis, Ras and Ras(G12V), a mutant that binds GTP, but is deficient in GTP hydrolysis were used to validate the assay [45]–[47]. Wildtype Ras displayed significantly higher GTPase activity compared to Ras G12V (data not shown). LRRK2 T1410A (G2019S) demonstrated a reduction in GTP hydrolysis when compared to LRRK2 wildtype, G2019S, and R1441C, a clinically observed mutation in the GTPase domain of LRRK2 (Figure 7C). Consistent with previous reports, R1441C showed decreased GTPase activity compared to both wildtype and G2019S [20], [21], [39], [48]. Furthermore, full length LRRK2 was immunoprecipitated from cells and assessed for GTP hydrolysis using thin-layer chromatography analysis. LRRK2 T1410A demonstrated a reduction in GTPase activity compared to LRRK2 wildtype, G2019S, and T1410D (Figure 7D). Interestingly, the kinase dead mutant, D1994N, also displayed reduced GTPase activity, that may be indicative of its inability to autophosphorylate. LRRK2 K1347A, which does not bind GTP [30], displayed activity similar to vector control background.

To further investigate the GTPase activity of T1410A, GTP binding assays using immobilized GTP were performed using transfected cell lysates containing LRRK2 wildtype, G2019S, K1347A, and T1410A(G2019S). LRRK2 T1410A (G2019S), wildtype, and G2019S all bound strongly to GTP (Figure 7E). K1347A, a mutation in the GTPase domain [30], inhibited GTP binding (Figure 7E). Moreover, addition of molar excess GTP competed for protein binding and eliminated pull-down of LRRK2 suggesting that the GTPase domain was bound to the GTP beads. GTP binding assays using GST-tagged LRRK2 wildtype, G2019S, R1441C, and T1410A recombinant protein displayed similar results suggesting that mutations do not affect GTP binding (data not shown). These results are consistent with previous findings that R1441C does not increase GTP binding even though GTPase activity was decreased [20], [21]. Together these data suggest that LRRK2 T1410A results in decreased GTP hydrolysis activity without a reduction in binding to GTP.

## Discussion

Our studies identified the motif F/Y-x-T-x-R/K to be the core sequence that LRRK2 prefers to phosphorylate, this was also reported in the consensus peptide substrate Nictide (RLGWWRFYTLRRARQGNTKQR) [29]. Our sequence preference differs from that of Nictide (WRFYTLRRA) with finding KKFKTQRRK as optimal. These differences suggest that there is low strength of selection of sites outside of the -2 and +2 positions. This is important in assessing the potential substrates of LRRK2 where less precedence should be given to these weaker positions. Interestingly we confirm that LRRK2 is a Thr preferring kinase, although rare, this preference for Thr over Ser has been reported for LRRK2 and other additional kinases [29], [49], [50].

Database searches using the consensus phosphorylation sequence revealed a number of potential downstream targets that may function in LRRK2 signaling. Of these, ATM, ULK1, Praja1, and MAP2 are neuronal and have potential relevance to PD. Moreover, peptidic sequences from them containing the identified LRRK2 consensus motif were phosphorylated. Mutations in ATM and LRRK2 can cause neurodegeneration and induce cell death respectively [51]–[53]. Moreover, insulin treatment has been shown to induce phosphorylation of 4E-BP1 in an ATM-dependent manner [54] and LRRK2 has been shown to phosphorylate 4E-BP1 [16], [55], indicating potential interplay between these pathways. ULK1 as a potential substrate suggests a mechanism by which LRRK2 could regulate autophagy. ULK1 plays a critical role during the activation of autophagy (reviewed in [56]) and knockdown of ULK1 suggests its involvement in starvation-dependent autophagy [57]. Knockdown of LRRK2 induces autophagic activity [58], and autophagy has been linked to LRRK2 mediated neurite retraction [59]. Moreover, ULK1 is phosphorylated although the kinase responsible remains unknown [60], [61]. MAP2 belongs to the microtubule-associated protein family and is found in neurons where it binds and stabilizes microtubules (reviewed in [62]). In addition, MAP2 interacts with actin filaments promoting neurite initiation [62], [63] and MAP2 phosphorylation alters binding to microtubules, the main mechanism of microtubule dynamic regulation (reviewed in [64]). Since LRRK2 affects neurite growth and actin cytoskeleton rearrangement [52], [59], [65], MAP2 as a potential substrate could explain such effects. Praja1 is an ubiquitin E3 ligase that is highly expressed in the brain [66], [67]. Praja1 has been shown to ubiquitinate Dlxin-1, a Dlx/Msx-binding protein that binds to the homeoprotein Dlx5 regulating its transcriptional activity [68]. Since the ubiquitin/proteasome pathway is associated with neurodegeneration [69] and E3 ligases are regulated by phosphorylation [70], LRRK2 may regulate Praja1, warranting further investigation of this as a potential substrate.

The identified consensus site intriguingly correlated with the sequence surrounding T1410 of LRRK2 itself. LRRK2 autophosphorylation has been thoroughly investigated and recently, three groups reported on LRRK2 autophosphorylation sites that overlap with this study [35]–[37]. The relevance of autophosphorylation on the ultimate function of LRRK2 remains unclear. The phosphomimetic T1491D appears to modulate kinase activity, although phosphorylation at other sites appears to have limited effects on LRRK2's ability to phosphorylate substrates [35]. Our findings also suggest that T1410 does not significantly alter LRRK2's kinase activity. Functionality of LRRK2 as a kinase has been demonstrated to require its dimerization [25]. Since T1410 lies in the Roc domain which has been shown to be involved in dimerization [39], it is conceivable that phosphorylation could modulate dimerization. Our studies indicate only a minor effect on dimerization is seen with the T1410D phosphomimetic mutant. Together the lack of significant effect of T1410 mutation on dimerization and kinase activity indicate that it does not have a major functional consequence on LRRK2's phosphorylating activity towards other substrates.

GTPase activity of LRRK2 is another key property that may have functional consequences. Although some studies have implicated GTPase activity in controlling kinase activity [21], [30], [38], [48], [71], [72], in yeast activity of the GTPase domain alone has been shown to influence toxicity [73]. Our studies indicated that mutation of T1410 can influence GTP hydrolysis, but with no gross effect on GTP binding, although we cannot discount more subtle effects on its binding properties [74]. The magnitude of these changes was small, but in line with changes observed in the pathogenic mutant R1441C in our studies and other reports [73]. It will be interesting to further confirm these results with directly phosphorylated T1410, as our studies have used a mutagenic approach that does not always fully recapitulate the physiological role of phosphorylation [75].

Identification of the LRRK2 G2019S phosphorylation motif has given insight to LRRK2's potential substrates and consequent pathophysiological function. These studies indicate that autophosphorylation may play a key role in regulating GTPase activity for functional consequence. Additionally, phosphorylation of other substrates identified in these studies suggest potential roles in disease. Uncovering the pathophysiological interaction network of LRRK2 is critical to understanding its contribution to PD pathogenesis.

## Supporting Information

Table S1 All phosphopeptides identified from LRRK2 G2019S. Recombinant LRRK2 G2019S was assayed for autophosphorylation followed by analysis by LC-MS/MS. This table lists each phosphopeptide identified from LRRK2 G2019S. Phosphopeptides identified from LRRK2 G2019S that are phosphorylated at [T/S]-x-[R/K[where x is any amino acid are bold. Peptide sequence, delta mass, charge, XCorr, site, and number of peptide to spectrum matches are listed. Phosphorylation is denoted by caret (∧), asterisk (*) denotes oxidation, and pound (#) denotes carbamidomethylation.
(0.12 MB DOC)

Figure S1 Overexposed Native-Blue PAGE. Overexposed Native-Blue PAGE shows the migration of high molecular weight, dimeric, and monomeric LRRK2. HEK-293T cells were transfected with empty vector or LRRK2 wildtype construct. Cell pellets were lysed by freeze/thaw cycles directly in PBS and 10 ug of protein lysate was loaded onto a native gel (3–12% Bis-Tris). LRRK2 complexes were immunoblotted with anti-HA antibody.
(0.29 MB TIF)
