# Supplementary material for: Identification and Characterization of a Leucine-Rich Repeat Kinase 2 (LRRK2) Consensus Phosphorylation Motif
Source: PLoS One. 2010 Oct 27;5(10):e13672. doi: 10.1371/journal.pone.0013672 (PMC2965117; doi:10.1371/journal.pone.0013672)
Supplement: Table S1 — All phosphopeptides identified from LRRK2 G2019S. Recombinant LRRK2 G2019S was assayed for autophosphorylation followed by analysis by LC-MS/MS. This table lists each phosphopeptide identified from LRRK2 G2019S. Phosphopeptides identified from LRRK2 G2019S that are phosphorylated at [T/S]-x-[R/K[where x is any amino acid are bold. Peptide sequence, delta mass, charge, XCorr, site, and number of peptide to spectrum matches are listed. Phosphorylation is denoted by caret (∧), asterisk (*) denotes oxidation, and pound (#) denotes carbamidomethylation. (0.12 MB DOC) [file pone.0013672.s001.doc]

**Supplemental Table S1. All phosphopeptides identified from LRRK2 G2019S.**

| Peptide | Δ Mass (Da) | Charge | XCorr | Residue(s) | Number of Peptide to Spectrum Matches |
| --- | --- | --- | --- | --- | --- |
| GFPAIRDYHFVNAT^EESDALAK | -0.0055 | 3 | 6.48 | Thr-1491 | 4 |
| GFPAIRDY^HFVNAT^EESDALAK | -0.0056 | 3 | 6.38 | Tyr-1485, Thr-1491 | 6 |
| ASSSPVILVGT^HLDVSDEK | -0.0025 | 2 | 5.15 | Thr-1452 | 16 |
| DYHFVNAT^EESDALAK | -0.0007 | 2 | 5.11 | Thr-1491 | 35 |
| **DLVLNVWDFAGREEFYS^THPHFM*T^QR** | **-0.0061** | **3** | **5.01** | **Ser-1403, Thr-1410** | **4** |
| **DLVLNVWDFAGREEFYST^HPHFM*T^QR** | **-0.0086** | **3** | **4.81** | **Thr-1404, Thr-1410** | **2** |
| ARAS^SSPVILVGT^HLDVSDEKQR | -0.0046 | 3 | 4.72 | Ser-1443, Thr-1452 | 6 |
| KSDLGM*QSAT^VGIDVKDWPIQIR | -0.0086 | 3 | 4.65 | Thr-1368 | 5 |
| LRKT^IINES^LNFK | -0.0016 | 3 | 4.62 | Thr-1503, Ser-1508 | 22 |
| RGFPAIRDYHFVNAT^EESDALAK | -0.0025 | 4 | 4.48 | Thr-1491 | 6 |
| LRKT^IINESLNFK | 0.0012 | 2 | 4.34 | Thr-1503 | 31 |
| ARASSS^PVILVGT^HLDVSDEKQR | 0.0029 | 4 | 4.32 | Ser-1445, Thr-1452 | 2 |
| **IM*AQILT^VKVEGCPK** | **-0.0031** | **2** | **4.11** | **Thr-1612** | **14** |
| SDLGM*QSAT^VGIDVK | 0.0005 | 2 | 4.08 | Thr-1368 | 10 |
| LTIPIS^QIAPDLILADLPR | 0.0033 | 3 | 4.05 | Ser-1853 | 2 |
| S^FPNEM*GKLSKIWDLPLDELHLNFDFK | -0.0082 | 3 | 3.97 | Ser-1283 | 1 |
| **IILS^ERKNVPIEFPVIDR** | **-0.0057** | **3** | **3.97** | **Ser-1536** | **2** |
| ASSSPVILVGT^HLDVSDEKQRK | 0.0014 | 3 | 3.93 | Thr-1452 | 3 |
| ASS^SPVILVGT^HLDVSDEK | -0.0031 | 2 | 3.92 | Ser-1444, Thr-1452 | 3 |
| ASSSPVILVGT^HLDVSDEKQR | -0.0068 | 2 | 3.87 | Thr-1452 | 13 |
| LT^IPISQIAPDLILADLPR | -0.0002 | 2 | 3.79 | Thr-1849 | 3 |
| SFPNEMGKLS^KIW*DLPLDELHLNFDFK | -0.0034 | 3 | 3.65 | Ser-1292 | 2 |
| **IMAQILT^VKVEGCPK** | **0.0005** | **2** | **3.55** | **Thr-1612** | **2** |
| ASSS^PVILVGT^HLDVSDEK | 0.0013 | 2 | 3.54 | Ser-1445, Thr-1452 | 1 |
| KACMS^KITK | -0.0002 | 2 | 3.50 | Ser-1467 | 2 |
| ASS^SPVILVGT^HLDVSDEKQR | -0.0021 | 3 | 3.43 | Ser-1444, Thr-1452 | 6 |
| ACMS^KITK | -0.0001 | 2 | 3.21 | Ser-1467 | 8 |
| ARAS^SSPVILVGT^HLDVSDEK | -0.0005 | 3 | 3.19 | Ser-1443, Thr-1452 | 1 |
| KT^IINESLNFK | 0.001 | 2 | 3.18 | Thr-1503 | 6 |
| MGIKT^SEGTPGFR | -0.0017 | 2 | 3.11 | Thr-2031 | 1 |
| T^IINESLNFK | 0.0008 | 2 | 3.07 | Thr-1503 | 7 |
| LM*IVGNT^GSGK | -0.0006 | 2 | 3.05 | Thr-1343 | 3 |
| DLVLNVWDFAGREEFY^S^THPHFM*TQR | -0.0114 | 4 | 3.04 | Tyr-1402, Ser-1403 | 1 |
| NYM*S^QYFK | 0.001 | 2 | 3.04 | Ser-1647 | 6 |
| **DLVLNVWDFAGREEFYSTHPHFM*T^QR** | **0.0008** | **3** | **2.97** | **Thr-1410** | **3** |
| LMIVGNT^GSGK | 0.0007 | 2 | 2.91 | Thr-1343 | 6 |
| MGIKT^SEGTPGFRAPEVAR | -0.0005 | 3 | 2.86 | Thr-2031 | 2 |
| **IFNKHTS^LR** | **0.0007** | **2** | **2.83** | **Ser-1913** | **7** |
| M*GIKT^SEGT^PGFRAPEVAR | 0.0001 | 3 | 2.75 | Thr-2031, Thr-2035 | 3 |
| IFNKHT^SLR | -0.0025 | 2 | 2.64 | Thr-1912 | 2 |
| M*TDSVTCLYCNSFSKQS^K | -0.0038 | 2 | 2.64 | Ser-2257 | 2 |
| ASLT^RT^LQHR | 0.0028 | 3 | 2.56 | Thr-1967, Thr-1969 | 6 |
| M*GIKT^SEGTPGFRAPEVAR | -0.0009 | 3 | 2.54 | Thr-2031 | 2 |
| KACMS^KIT^K | 0.0008 | 2 | 2.50 | Ser-1467, Thr-1470 | 5 |
| ASSSPVILVGTHLDVS^DEKQRK | -0.0033 | 4 | 2.46 | Ser-1457 | 2 |
| DLVLNVWDFAGREEFYS^THPHFM*TQR | -0.017 | 4 | 2.42 | Ser-1403 | 2 |
| SFPNEM*GKLS^KIWDLPLDELHLNFDFK | 0.0042 | 4 | 2.30 | Ser-1292 | 6 |
| KACM*S^KITK | -0.0005 | 2 | 2.27 | Ser-1467 | 2 |
| TTLLQQLM*KT^K | 0.0009 | 2 | 2.22 | Thr-1357 | 9 |
| SFPNEM*GKLS^K | -0.0011 | 2 | 2.22 | Ser-1292 | 7 |
| **LMIVGNTGS^GK** | **-0.0002** | **2** | **2.17** | **Ser-1345** | **2** |
| NYMS^QYFK | 0 | 2 | 2.11 | Ser-1647 | 2 |
| MGIKT^SEGT^PGFR | 0.0008 | 2 | 2.05 | Thr-2031, Thr-2035 | 3 |
| EEFYST^HPHFMTQR | -0.0015 | 3 | 2.04 | Thr-1404 | 2 |
| ACMS^KIT^KELLNK | 0.0015 | 3 | 2.01 | Ser-1467, Thr-1470 | 6 |
| **EEFYS^THPHFM*T^QR** | **-0.0002** | **3** | **1.95** | **Ser-1403, Thr-1410** | **2** |
| **IM*AQILT^VKVEGC#PK** | **0.004** | **3** | **1.94** | **Thr-1612** | **2** |
| AS^SSPVILVGT^HLDVSDEKQRK | -0.0045 | 3 | 1.89 | Ser-1443, Thr-1452 | 1 |
| **EEFYSTHPHFMT^QR** | **0.0007** | **3** | **1.81** | **Thr-1410** | **4** |
| **EEFYSTHPHFM*T^QR** | **-0.0033** | **2** | **1.81** | **Thr-1410** | **4** |
| MGIKT^SEGT^PGFRAPEVAR | -0.0035 | 3 | 1.79 | Thr-2031, Thr-2035 | 1 |
| SFPNEMGKLS^K | 0.0007 | 2 | 1.76 | Ser-1292 | 10 |
| EEFYS^T^HPHFM*TQR | 0.002 | 3 | 1.70 | Ser-1403, Thr-1404 | 2 |
| M*GIKT^S^EGTPGFRAPEVAR | 0.0012 | 3 | 1.64 | Thr-2031, Ser-2032 | 1 |
| MRRT^SVE | 0.0018 | 2 | 1.61 | Thr-2524 | 13 |
| KAVPY^NR | 0.0009 | 2 | 1.49 | Tyr-1332 | 1 |
| LHLS^HNK | 0.0007 | 2 | 1.48 | Ser-1253 | 2 |
| **EEFYS^THPHFMT^QR** | **0.0025** | **3** | **1.27** | **Ser-1403, Thr-1410** | **1** |
| ASSSPVILVGTHLDVS^DEK | -0.0057 | 3 | 1.08 | Ser-1457 | 1 |

Recombinant LRRK2 G2019S was assayed for autophosphorylation followed by analysis by LC-MS/MS. This table lists each phosphopeptide identified from LRRK2 G2019S. Phosphopeptides identified from LRRK2 G2019S that are phosphorylated at [T/S]-x-[R/K[ where x is any amino acid are bold. Peptide sequence, delta mass, charge, XCorr, site, and number of peptide to spectrum matches are listed. Phosphorylation is denoted by caret (^), asterisk (*) denotes oxidation, and pound (#) denotes carbamidomethylation.
