# Supplementary figures and images for: Identification and Characterization of a Leucine-Rich Repeat Kinase 2 (LRRK2) Consensus Phosphorylation Motif
Source: PLoS One. 2010 Oct 27;5(10):e13672. doi: 10.1371/journal.pone.0013672 (PMC2965117; doi:10.1371/journal.pone.0013672)

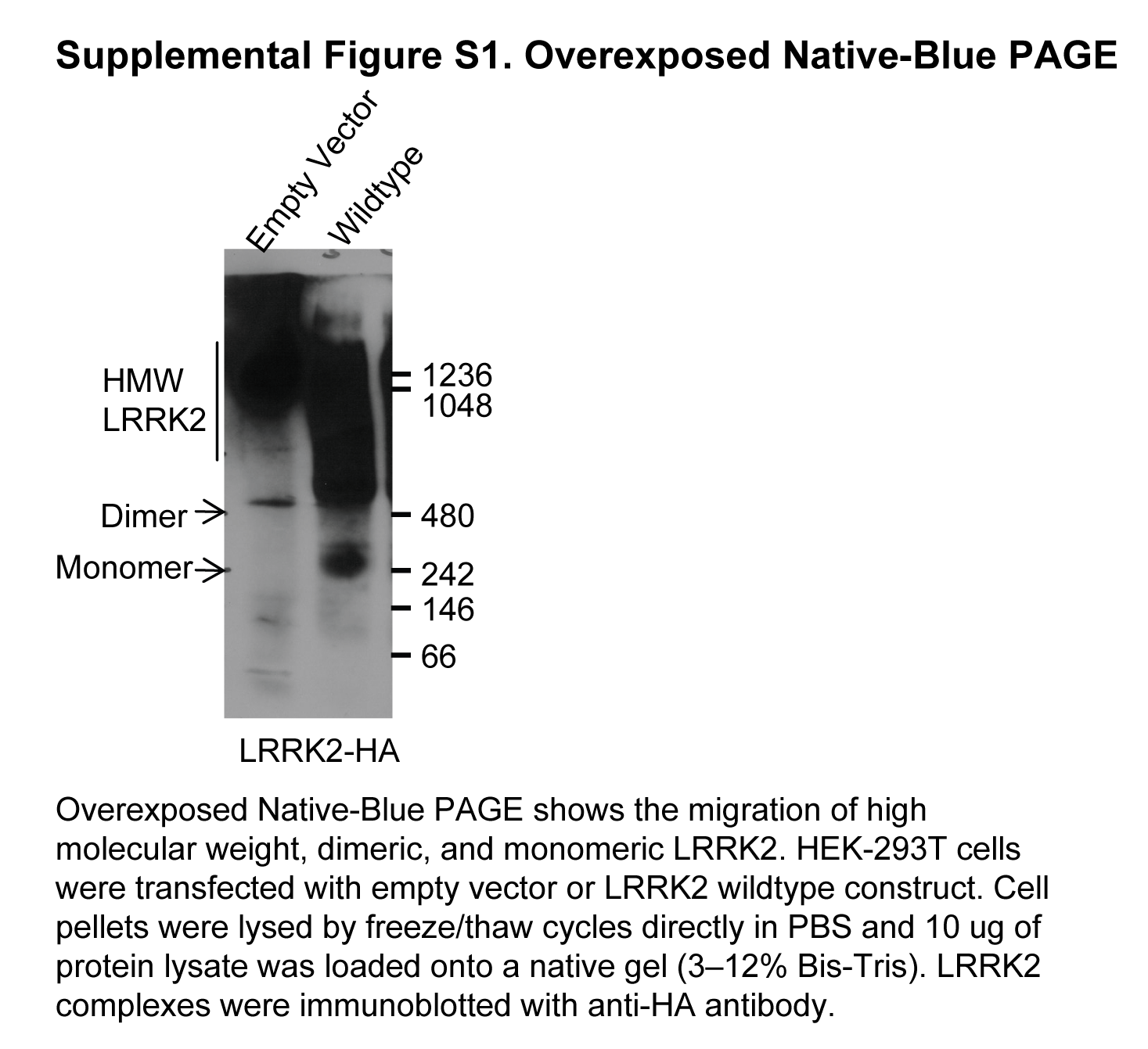

Supplement: Figure S1 — Overexposed Native-Blue PAGE. Overexposed Native-Blue PAGE shows the migration of high molecular weight, dimeric, and monomeric LRRK2. HEK-293T cells were transfected with empty vector or LRRK2 wildtype construct. Cell pellets were lysed by freeze/thaw cycles directly in PBS and 10 ug of protein lysate was loaded onto a native gel (3–12% Bis-Tris). LRRK2 complexes were immunoblotted with anti-HA antibody. (0.29 MB TIF) [file pone.0013672.s002.tif]
